# Crystal structure of (2-bromo­methyl-1-phenyl­sulfonyl-1*H*-indol-3-yl)(phen­yl)methanone

**DOI:** 10.1107/S2056989014028084

**Published:** 2015-01-03

**Authors:** M. Umadevi, V. Saravanan, R. Yamuna, A. K. Mohanakrishnan, G. Chakkaravarthi

**Affiliations:** a Research and Development Centre, Bharathiyar University, Coimbatore 641 046, India; b Department of Chemistry, Pallavan College of Engineering, Kanchipuram 631 502, India; c Department of Organic Chemistry, University of Madras, Guindy Campus, Chennai 600 025, India; d Department of Sciences, Chemistry and Materials Research Lab, Amrita Vishwa Vidyapeetham University, Ettimadai, Coimbatore 641 112, India; e Department of Physics, CPCL Polytechnic College, Chennai 600 068, India

**Keywords:** crystal structure, indole, phenyl­sulfon­yl, bromo­meth­yl, (phen­yl)methanone, intramolecular hydrogen bonds

## Abstract

In the title compound, C_22_H_16_BrNO_3_S, the phenyl rings make dihedral angles of 84.81 (16) and 61.67 (17)° with the indole ring system (r.m.s. deviation = 0.012 Å), while the phenyl rings are inclined to one another by 69.5 (2)°. The mol­ecular structure is stabilized by weak intra­molecular C—H⋯O hydrogen bonds. The sulfonyl S atom has a distorted tetra­hedral configuration. In the crystal, there are no significant inter­molecular inter­actions present.

## Related literature   

For the various biological properties of indole derivatives, see: Andreani *et al.* (2001 ▸); Bassindale (1984 ▸); Grinev *et al.* (1984 ▸); Porter *et al.* (1977 ▸); Rodriguez *et al.* (1985 ▸); Singh *et al.* (2000 ▸); Sundberg (1996 ▸). For the Thorpe–Ingold effect, see: Bassindale (1984 ▸). For the syntheses and crystal structures of similar compounds, see: Chakkaravarthi *et al.* (2008 ▸, 2009 ▸); Umadevi *et al.* (2013 ▸, 2014 ▸). For details concerning the Cambridge Structural Database, see: Groom & Allen (2014 ▸).
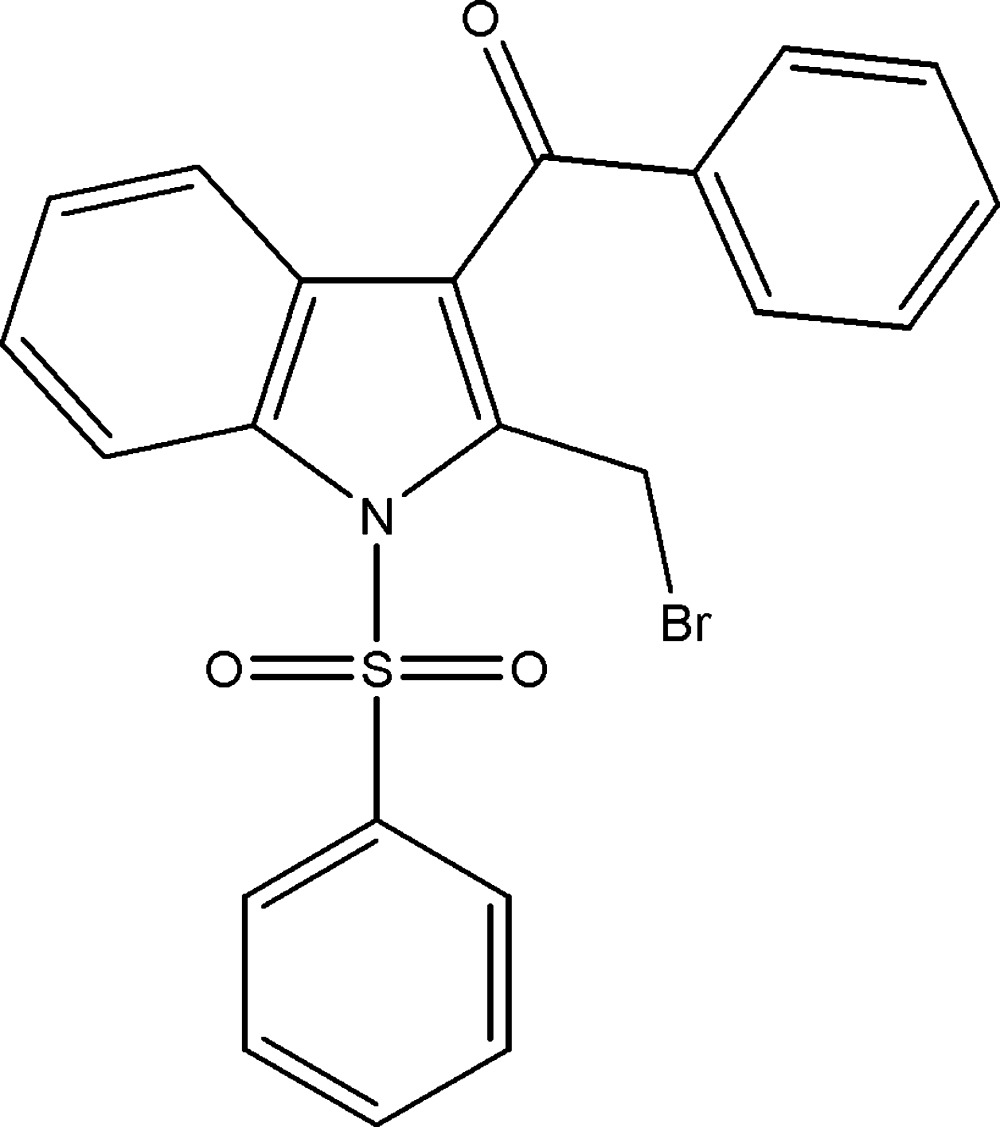


## Experimental   

### Crystal data  

C_22_H_16_BrNO_3_S
*M* *_r_* = 454.33
Monoclinic, 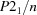

*a* = 10.3629 (5) Å
*b* = 13.4156 (7) Å
*c* = 14.1777 (8) Å
β = 92.864 (2)°
*V* = 1968.59 (18) Å^3^
*Z* = 4
Mo *K*α radiation
μ = 2.22 mm^−1^
*T* = 295 K
0.26 × 0.22 × 0.20 mm

### Data collection  

Bruker Kappa APEXII CCD diffractometer
Absorption correction: multi-scan (*SADABS*; Sheldrick, 1996 ▸) *T*_min_ = 0.596, *T*_max_ = 0.665
34711 measured reflections
5017 independent reflections
3002 reflections with *I* > 2σ(*I*)
*R*_int_ = 0.056

### Refinement  

*R*[*F* ^2^ > 2σ(*F* ^2^)] = 0.045
*wR*(*F* ^2^) = 0.146
*S* = 1.06
5017 reflections
253 parameters
H-atom parameters constrained
Δρ_max_ = 0.38 e Å^−3^
Δρ_min_ = −0.97 e Å^−3^

### 

Data collection: *APEX2* (Bruker, 2004 ▸); cell refinement: *SAINT* (Bruker, 2004 ▸); data reduction: *SAINT*; program(s) used to solve structure: *SHELXS97* (Sheldrick, 2008 ▸); program(s) used to refine structure: *SHELXL97* (Sheldrick, 2008 ▸); molecular graphics: *PLATON* (Spek, 2009 ▸); software used to prepare material for publication: *SHELXL97* and *PLATON* (Spek, 2009 ▸).

## Supplementary Material

Crystal structure: contains datablock(s) I, global. DOI: 10.1107/S2056989014028084/su5047sup1.cif

Structure factors: contains datablock(s) I. DOI: 10.1107/S2056989014028084/su5047Isup2.hkl

Supporting information file. DOI: 10.1107/S2056989014028084/su5047Isup3.cml

**Fig. 1.**
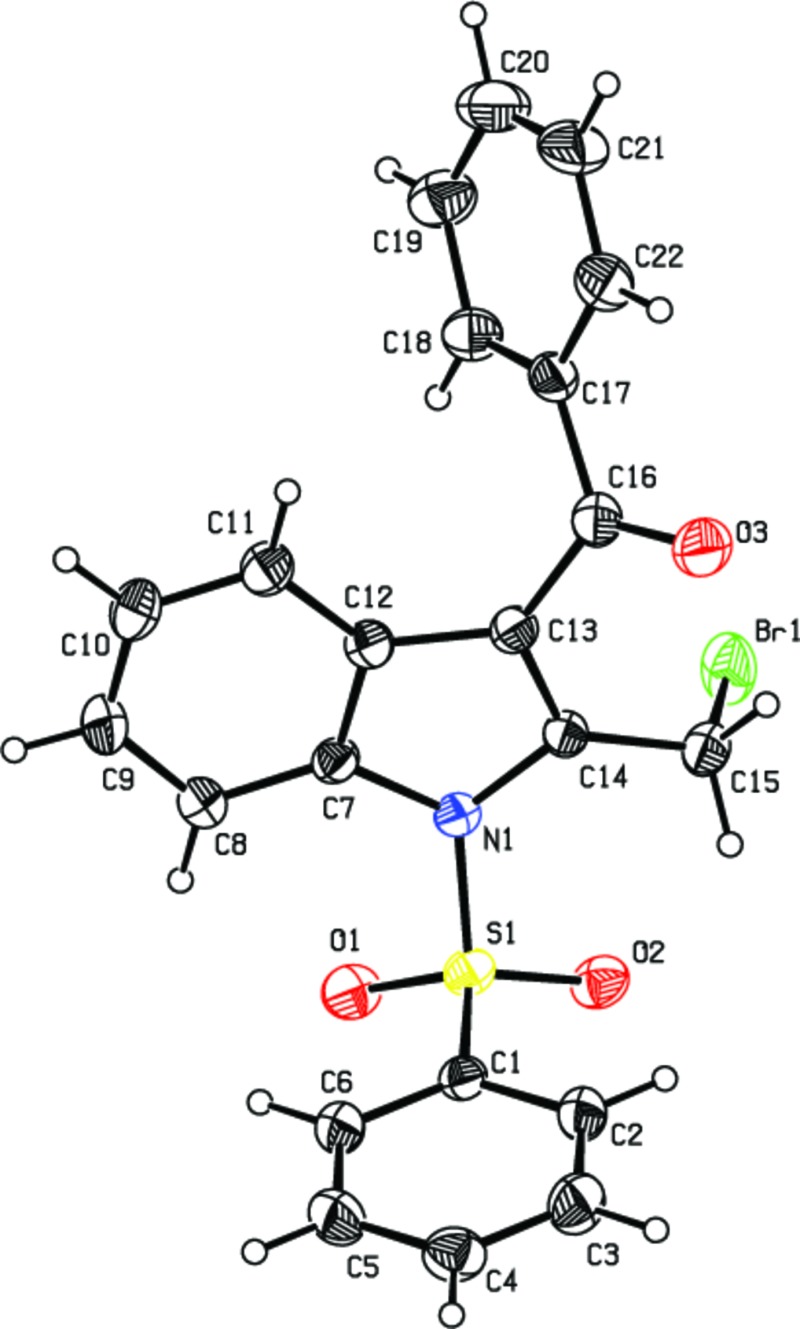
The molecular structure of the title compound, showing the atom labelling. Displacement ellipsoids are drawn at the 30% probability level.

**Fig. 2.**
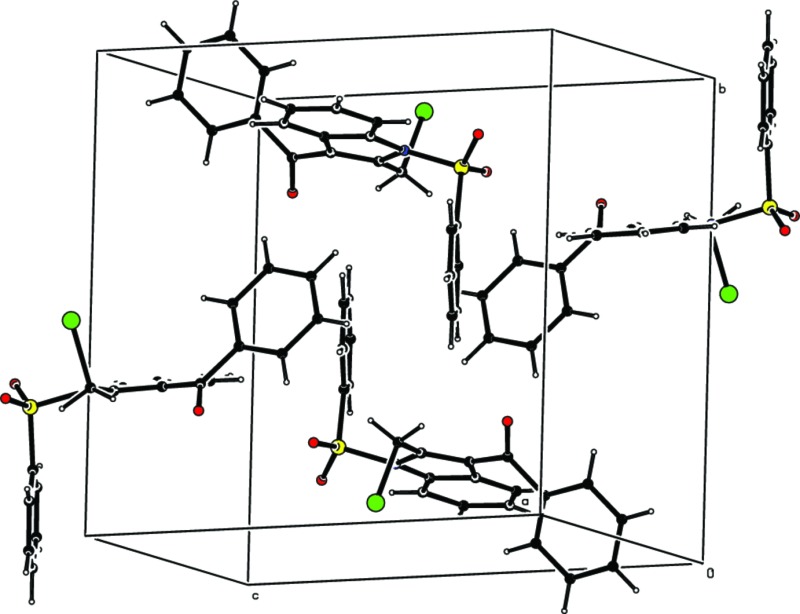
A view approximately along the a axis of the crystal packing of the title compound.

. DOI: 10.1107/S2056989014028084/su5047fig1.tif
The mol­ecular structure of the title compound, showing the atom labelling. Displacement ellipsoids are drawn at the 30% probability level.

a . DOI: 10.1107/S2056989014028084/su5047fig2.tif
A view approximately along the *a* axis of the crystal packing of the title compound.

CCDC reference: 1040945

Additional supporting information:  crystallographic information; 3D view; checkCIF report

## Figures and Tables

**Table 1:** Hydrogen-bond geometry (, )

*D*H*A*	*D*H	H*A*	*D* *A*	*D*H*A*
C8H8O1	0.93	2.38	2.953(4)	120
C15H15*A*O2	0.97	2.22	2.808(4)	118
C15H15*B*O3	0.97	2.38	3.062(5)	127

## supplementary crystallographic information

### S1. Synthesis and crystallization

To a solution of (2-methyl-1-(phenyl­sulfonyl)-1H-indol-3-yl)(phenyl)­methanone (1 g, 2.67 mmol) in dry carbon tetra­chloride (75 mL), AIBN (0.05 g) and finely powdered NBS (0.61 g, 3.47 mmol) were added. The reaction mixture was refluxed for 2 h and cooled to room temperature. The floated succinimide was filtered off and washed with carbon tetra­chloride (15 mL). The combined filtrate was concentrated in vacuo to afford title compound (1.12 g, 92%) as a colourless solid (156-158°C), suitable for X-ray diffraction.

### S1.1. Refinement

Crystal data, data collection and structure refinement details are summarized in Table 2. C-bound H atoms were included in calculated positions and treated as riding atoms: C—H = 0.93 - 0.97 Å with U_iso_(H) = 1.5U_eq_(C) for methyl H atoms and = 1.2U_eq_(C) for other H atoms.

### S2. Structural commentary

Indole derivatives are known to exhibit anti­bacterial, anti­fungal (Singh *et al.*, 2000), anti­tumour (Andreani *et al.*, 2001), anti­depressant (Grinev *et al.*, 1984), anti-inflammatory (Rodriguez *et al.*, 1985) and physiological (Porter *et al.*, 1977; Sundberg, 1996) properties. In recent years, we have reported the synthesis and crystal structures of a number of such compounds (Chakkaravarthi *et al.*, 2008, 2009; Umadevi *et al.*, 2013, 2014). We report herein on the synthesis and crystal structure of the title compound, a new 1-(phenyl­sulfonyl)-1*H*-indole derivative.

The molecular structure of the title compound is shown in Fig. 1. The phenyl rings (C1—C6) and (C17—C22) make the dihedral angles of 84.81 (16)° and 61.67 (17)°, respectively, with the indole ring system (N1/C7—C14). The phenyl rings (C1—C6) and (C17—C22) are inclined at an angle of 69.5 (2)° with respect to each other. As a result of electron–withdrawing character of the phenyl­sulfonyl group, the bond lengths N1—C7 = 1.415 (4) Å and N1—C14 = 1.409 (4) Å are longer than the mean value of 1.355 (14) Å observed for similar compounds in the Cambridge Structural Database (Groom & Allen *et al.*, 2014). Atom S1 of the sulfonyl group has a distorted tetra­hedral configuration. The widening of angle O1—S1—O2 [120.70 (15)°] and the narrowing of angle N1—S1—C1 [104.71 (14)°] from the ideal tetra­hedral value are attributed to the Thorpe–Ingold effect (Bassindale, 1984). The molecular structure is stabilized by weak C—H···O intra­molecular hydrogen bonds (Table 1).

In the crystal, there are no significant inter­molecular inter­actions present, Fig. 2.

### Crystal data

**Table:**

C_22_H_16_BrNO_3_S	*F*(000) = 920
*M**_r_* = 454.33	*D*_x_ = 1.533 Mg m^−^^3^
Monoclinic, *P*2_1_/*n*	Mo *K*α radiation, λ = 0.71073 Å
Hall symbol: -P 2yn	Cell parameters from 8313 reflections
*a* = 10.3629 (5) Å	θ = 2.3–23.1°
*b* = 13.4156 (7) Å	µ = 2.22 mm^−^^1^
*c* = 14.1777 (8) Å	*T* = 295 K
β = 92.864 (2)°	Block, colourless
*V* = 1968.59 (18) Å^3^	0.26 × 0.22 × 0.20 mm
*Z* = 4	

### Data collection

**Table:**

Bruker Kappa APEXII CCD diffractometer	5017 independent reflections
Radiation source: fine-focus sealed tube	3002 reflections with *I* > 2σ(*I*)
Graphite monochromator	*R*_int_ = 0.056
ω and φ scan	θ_max_ = 28.6°, θ_min_ = 2.1°
Absorption correction: multi-scan (*SADABS*; Sheldrick, 1996)	*h* = −13→13
*T*_min_ = 0.596, *T*_max_ = 0.665	*k* = −18→18
34711 measured reflections	*l* = −19→14

### Refinement

**Table:**

Refinement on *F*^2^	Primary atom site location: structure-invariant direct methods
Least-squares matrix: full	Secondary atom site location: difference Fourier map
*R*[*F*^2^ > 2σ(*F*^2^)] = 0.045	Hydrogen site location: inferred from neighbouring sites
*wR*(*F*^2^) = 0.146	H-atom parameters constrained
*S* = 1.06	*w* = 1/[σ^2^(*F*_o_^2^) + (0.0515*P*)^2^ + 1.6826*P*] where *P* = (*F*_o_^2^ + 2*F*_c_^2^)/3
5017 reflections	(Δ/σ)_max_ < 0.001
253 parameters	Δρ_max_ = 0.38 e Å^−^^3^
0 restraints	Δρ_min_ = −0.97 e Å^−^^3^

### Special details

**Table:**

Geometry. All e.s.d.'s (except the e.s.d. in the dihedral angle between two l.s. planes) are estimated using the full covariance matrix. The cell e.s.d.'s are taken into account individually in the estimation of e.s.d.'s in distances, angles and torsion angles; correlations between e.s.d.'s in cell parameters are only used when they are defined by crystal symmetry. An approximate (isotropic) treatment of cell e.s.d.'s is used for estimating e.s.d.'s involving l.s. planes.
Refinement. Refinement of *F*^2^ against ALL reflections. The weighted *R*-factor *wR* and goodness of fit *S* are based on *F*^2^, conventional *R*-factors *R* are based on *F*, with *F* set to zero for negative *F*^2^. The threshold expression of *F*^2^ > σ(*F*^2^) is used only for calculating *R*-factors(gt) *etc*. and is not relevant to the choice of reflections for refinement. *R*-factors based on *F*^2^ are statistically about twice as large as those based on *F*, and *R*- factors based on ALL data will be even larger.

### Fractional atomic coordinates and isotropic or equivalent isotropic displacement parameters (Å^2^)

**Table:**

	*x*	*y*	*z*	*U*_iso_*/*U*_eq_	
C1	0.4019 (3)	0.3474 (2)	0.6542 (2)	0.0375 (7)	
C2	0.4866 (3)	0.4214 (3)	0.6300 (3)	0.0476 (8)	
H2	0.5662	0.4056	0.6064	0.057*	
C3	0.4506 (4)	0.5192 (3)	0.6415 (3)	0.0609 (11)	
H3	0.5067	0.5702	0.6262	0.073*	
C4	0.3325 (4)	0.5421 (3)	0.6754 (3)	0.0571 (10)	
H4	0.3093	0.6084	0.6830	0.069*	
C5	0.2489 (4)	0.4687 (3)	0.6980 (3)	0.0583 (10)	
H5	0.1688	0.4850	0.7205	0.070*	
C6	0.2829 (3)	0.3700 (3)	0.6876 (3)	0.0493 (9)	
H6	0.2262	0.3194	0.7030	0.059*	
C7	0.2714 (3)	0.1711 (2)	0.5076 (2)	0.0374 (7)	
C8	0.1644 (3)	0.1550 (3)	0.5608 (3)	0.0479 (8)	
H8	0.1702	0.1566	0.6264	0.057*	
C9	0.0499 (3)	0.1366 (3)	0.5115 (3)	0.0563 (10)	
H9	−0.0238	0.1253	0.5447	0.068*	
C10	0.0403 (3)	0.1341 (3)	0.4142 (3)	0.0587 (10)	
H10	−0.0394	0.1210	0.3836	0.070*	
C11	0.1456 (3)	0.1506 (3)	0.3619 (3)	0.0500 (9)	
H11	0.1382	0.1500	0.2962	0.060*	
C12	0.2641 (3)	0.1684 (2)	0.4096 (2)	0.0381 (7)	
C13	0.3916 (3)	0.1867 (2)	0.3780 (2)	0.0382 (7)	
C14	0.4730 (3)	0.2001 (2)	0.4550 (2)	0.0365 (7)	
C15	0.6139 (3)	0.2080 (3)	0.4556 (3)	0.0461 (8)	
H15A	0.6444	0.2520	0.5062	0.055*	
H15B	0.6393	0.2362	0.3963	0.055*	
C16	0.4326 (3)	0.1888 (3)	0.2792 (2)	0.0451 (8)	
C17	0.3870 (3)	0.1104 (3)	0.2123 (2)	0.0437 (8)	
C18	0.3509 (4)	0.0169 (3)	0.2420 (3)	0.0511 (9)	
H18	0.3486	0.0030	0.3062	0.061*	
C19	0.3180 (4)	−0.0558 (3)	0.1765 (3)	0.0663 (11)	
H19	0.2937	−0.1189	0.1962	0.080*	
C20	0.3213 (5)	−0.0347 (4)	0.0816 (3)	0.0753 (13)	
H20	0.2992	−0.0839	0.0375	0.090*	
C21	0.3566 (5)	0.0572 (4)	0.0519 (3)	0.0765 (14)	
H21	0.3579	0.0709	−0.0124	0.092*	
C22	0.3903 (4)	0.1298 (3)	0.1165 (3)	0.0595 (10)	
H22	0.4155	0.1924	0.0960	0.071*	
N1	0.4022 (2)	0.1883 (2)	0.53652 (18)	0.0379 (6)	
O1	0.3769 (2)	0.16500 (18)	0.70726 (16)	0.0523 (6)	
O2	0.5868 (2)	0.21867 (18)	0.65144 (17)	0.0500 (6)	
O3	0.5064 (3)	0.2531 (2)	0.25507 (19)	0.0673 (8)	
S1	0.44977 (8)	0.22304 (6)	0.64628 (6)	0.0400 (2)	
Br1	0.69179 (4)	0.07623 (3)	0.47354 (4)	0.06721 (18)	

### Atomic displacement parameters (Å^2^)

**Table:**

	*U*^11^	*U*^22^	*U*^33^	*U*^12^	*U*^13^	*U*^23^
C1	0.0397 (16)	0.0389 (17)	0.0338 (16)	−0.0043 (14)	0.0000 (13)	−0.0028 (13)
C2	0.0439 (18)	0.047 (2)	0.053 (2)	−0.0059 (15)	0.0085 (16)	−0.0017 (16)
C3	0.065 (3)	0.043 (2)	0.075 (3)	−0.0114 (19)	0.011 (2)	−0.0001 (19)
C4	0.072 (3)	0.041 (2)	0.059 (2)	0.0008 (19)	0.006 (2)	−0.0039 (18)
C5	0.055 (2)	0.055 (2)	0.066 (3)	0.0060 (19)	0.0141 (19)	−0.010 (2)
C6	0.048 (2)	0.047 (2)	0.054 (2)	−0.0087 (16)	0.0107 (16)	−0.0060 (17)
C7	0.0337 (15)	0.0378 (17)	0.0403 (18)	−0.0005 (13)	−0.0024 (13)	−0.0039 (14)
C8	0.0427 (18)	0.057 (2)	0.045 (2)	−0.0028 (16)	0.0077 (15)	−0.0062 (17)
C9	0.0365 (18)	0.067 (3)	0.067 (3)	−0.0023 (17)	0.0126 (17)	−0.008 (2)
C10	0.0331 (18)	0.077 (3)	0.065 (3)	−0.0014 (18)	−0.0076 (17)	−0.005 (2)
C11	0.0391 (18)	0.068 (2)	0.0424 (19)	0.0001 (17)	−0.0070 (15)	−0.0012 (17)
C12	0.0361 (16)	0.0366 (17)	0.0413 (18)	0.0045 (13)	−0.0005 (13)	−0.0007 (14)
C13	0.0371 (16)	0.0370 (17)	0.0404 (18)	−0.0005 (13)	0.0003 (13)	−0.0024 (14)
C14	0.0338 (15)	0.0350 (17)	0.0409 (18)	−0.0013 (13)	0.0038 (13)	−0.0030 (13)
C15	0.0409 (18)	0.046 (2)	0.051 (2)	−0.0076 (15)	0.0020 (15)	0.0005 (16)
C16	0.0419 (18)	0.048 (2)	0.045 (2)	−0.0019 (15)	0.0023 (15)	0.0027 (16)
C17	0.0375 (17)	0.054 (2)	0.0398 (19)	0.0025 (15)	0.0026 (14)	−0.0045 (16)
C18	0.057 (2)	0.049 (2)	0.047 (2)	0.0036 (17)	−0.0020 (16)	−0.0013 (17)
C19	0.073 (3)	0.053 (2)	0.072 (3)	0.003 (2)	−0.003 (2)	−0.011 (2)
C20	0.081 (3)	0.080 (3)	0.065 (3)	0.006 (3)	−0.004 (2)	−0.028 (3)
C21	0.082 (3)	0.108 (4)	0.039 (2)	−0.003 (3)	−0.001 (2)	−0.016 (2)
C22	0.064 (2)	0.070 (3)	0.045 (2)	−0.005 (2)	0.0068 (18)	0.002 (2)
N1	0.0352 (13)	0.0414 (15)	0.0370 (14)	−0.0019 (11)	0.0005 (11)	−0.0066 (12)
O1	0.0671 (16)	0.0479 (15)	0.0419 (14)	−0.0081 (12)	0.0011 (11)	0.0074 (11)
O2	0.0431 (13)	0.0536 (15)	0.0518 (14)	0.0059 (11)	−0.0122 (11)	−0.0047 (12)
O3	0.081 (2)	0.0693 (19)	0.0529 (16)	−0.0295 (15)	0.0130 (14)	−0.0012 (14)
S1	0.0418 (4)	0.0408 (5)	0.0368 (4)	−0.0016 (3)	−0.0048 (3)	−0.0001 (3)
Br1	0.0408 (2)	0.0659 (3)	0.0953 (4)	0.01197 (18)	0.0068 (2)	0.0010 (2)

### Geometric parameters (Å, º)

**Table:**

C1—C6	1.378 (5)	C13—C14	1.357 (4)
C1—C2	1.379 (5)	C13—C16	1.483 (5)
C1—S1	1.746 (3)	C14—N1	1.409 (4)
C2—C3	1.377 (5)	C14—C15	1.463 (4)
C2—H2	0.9300	C15—Br1	1.955 (4)
C3—C4	1.371 (5)	C15—H15A	0.9700
C3—H3	0.9300	C15—H15B	0.9700
C4—C5	1.360 (5)	C16—O3	1.213 (4)
C4—H4	0.9300	C16—C17	1.479 (5)
C5—C6	1.380 (5)	C17—C18	1.381 (5)
C5—H5	0.9300	C17—C22	1.385 (5)
C6—H6	0.9300	C18—C19	1.378 (5)
C7—C8	1.388 (4)	C18—H18	0.9300
C7—C12	1.389 (4)	C19—C20	1.376 (7)
C7—N1	1.415 (4)	C19—H19	0.9300
C8—C9	1.370 (5)	C20—C21	1.360 (7)
C8—H8	0.9300	C20—H20	0.9300
C9—C10	1.378 (5)	C21—C22	1.370 (6)
C9—H9	0.9300	C21—H21	0.9300
C10—C11	1.368 (5)	C22—H22	0.9300
C10—H10	0.9300	N1—S1	1.675 (3)
C11—C12	1.392 (4)	O1—S1	1.411 (2)
C11—H11	0.9300	O2—S1	1.419 (2)
C12—C13	1.438 (4)		
			
C6—C1—C2	121.3 (3)	C13—C14—N1	108.5 (3)
C6—C1—S1	119.6 (3)	C13—C14—C15	126.4 (3)
C2—C1—S1	119.1 (3)	N1—C14—C15	124.3 (3)
C3—C2—C1	118.5 (3)	C14—C15—Br1	109.9 (2)
C3—C2—H2	120.8	C14—C15—H15A	109.7
C1—C2—H2	120.8	Br1—C15—H15A	109.7
C4—C3—C2	120.4 (4)	C14—C15—H15B	109.7
C4—C3—H3	119.8	Br1—C15—H15B	109.7
C2—C3—H3	119.8	H15A—C15—H15B	108.2
C5—C4—C3	120.8 (4)	O3—C16—C17	120.6 (3)
C5—C4—H4	119.6	O3—C16—C13	119.6 (3)
C3—C4—H4	119.6	C17—C16—C13	119.7 (3)
C4—C5—C6	120.0 (4)	C18—C17—C22	119.4 (3)
C4—C5—H5	120.0	C18—C17—C16	122.2 (3)
C6—C5—H5	120.0	C22—C17—C16	118.2 (3)
C1—C6—C5	119.0 (3)	C19—C18—C17	119.9 (4)
C1—C6—H6	120.5	C19—C18—H18	120.1
C5—C6—H6	120.5	C17—C18—H18	120.1
C8—C7—C12	122.3 (3)	C20—C19—C18	119.8 (4)
C8—C7—N1	130.4 (3)	C20—C19—H19	120.1
C12—C7—N1	107.3 (3)	C18—C19—H19	120.1
C9—C8—C7	116.5 (3)	C21—C20—C19	120.6 (4)
C9—C8—H8	121.7	C21—C20—H20	119.7
C7—C8—H8	121.7	C19—C20—H20	119.7
C8—C9—C10	122.2 (3)	C20—C21—C22	120.1 (4)
C8—C9—H9	118.9	C20—C21—H21	120.0
C10—C9—H9	118.9	C22—C21—H21	120.0
C11—C10—C9	121.3 (3)	C21—C22—C17	120.3 (4)
C11—C10—H10	119.4	C21—C22—H22	119.9
C9—C10—H10	119.4	C17—C22—H22	119.9
C10—C11—C12	118.2 (3)	C14—N1—C7	108.1 (2)
C10—C11—H11	120.9	C14—N1—S1	126.2 (2)
C12—C11—H11	120.9	C7—N1—S1	123.2 (2)
C7—C12—C11	119.6 (3)	O1—S1—O2	120.70 (15)
C7—C12—C13	107.6 (3)	O1—S1—N1	105.92 (14)
C11—C12—C13	132.8 (3)	O2—S1—N1	106.48 (14)
C14—C13—C12	108.4 (3)	O1—S1—C1	109.02 (15)
C14—C13—C16	124.1 (3)	O2—S1—C1	108.82 (15)
C12—C13—C16	127.5 (3)	N1—S1—C1	104.71 (14)
			
C6—C1—C2—C3	1.1 (5)	O3—C16—C17—C18	152.3 (4)
S1—C1—C2—C3	−176.4 (3)	C13—C16—C17—C18	−25.4 (5)
C1—C2—C3—C4	−0.7 (6)	O3—C16—C17—C22	−22.8 (5)
C2—C3—C4—C5	−0.1 (6)	C13—C16—C17—C22	159.5 (3)
C3—C4—C5—C6	0.5 (6)	C22—C17—C18—C19	−0.2 (5)
C2—C1—C6—C5	−0.8 (5)	C16—C17—C18—C19	−175.3 (4)
S1—C1—C6—C5	176.7 (3)	C17—C18—C19—C20	−0.1 (6)
C4—C5—C6—C1	0.0 (6)	C18—C19—C20—C21	0.0 (7)
C12—C7—C8—C9	−0.4 (5)	C19—C20—C21—C22	0.5 (8)
N1—C7—C8—C9	−177.5 (3)	C20—C21—C22—C17	−0.8 (7)
C7—C8—C9—C10	−0.1 (6)	C18—C17—C22—C21	0.7 (6)
C8—C9—C10—C11	−0.3 (7)	C16—C17—C22—C21	176.0 (4)
C9—C10—C11—C12	1.1 (6)	C13—C14—N1—C7	−2.4 (4)
C8—C7—C12—C11	1.2 (5)	C15—C14—N1—C7	−172.9 (3)
N1—C7—C12—C11	178.9 (3)	C13—C14—N1—S1	−164.8 (2)
C8—C7—C12—C13	−179.2 (3)	C15—C14—N1—S1	24.7 (4)
N1—C7—C12—C13	−1.5 (3)	C8—C7—N1—C14	179.9 (4)
C10—C11—C12—C7	−1.5 (5)	C12—C7—N1—C14	2.4 (3)
C10—C11—C12—C13	179.0 (4)	C8—C7—N1—S1	−17.1 (5)
C7—C12—C13—C14	0.0 (4)	C12—C7—N1—S1	165.4 (2)
C11—C12—C13—C14	179.5 (4)	C14—N1—S1—O1	−158.0 (3)
C7—C12—C13—C16	178.5 (3)	C7—N1—S1—O1	42.1 (3)
C11—C12—C13—C16	−1.9 (6)	C14—N1—S1—O2	−28.4 (3)
C12—C13—C14—N1	1.5 (4)	C7—N1—S1—O2	171.7 (2)
C16—C13—C14—N1	−177.1 (3)	C14—N1—S1—C1	86.8 (3)
C12—C13—C14—C15	171.7 (3)	C7—N1—S1—C1	−73.1 (3)
C16—C13—C14—C15	−6.9 (5)	C6—C1—S1—O1	−21.0 (3)
C13—C14—C15—Br1	−91.4 (4)	C2—C1—S1—O1	156.5 (3)
N1—C14—C15—Br1	77.3 (4)	C6—C1—S1—O2	−154.5 (3)
C14—C13—C16—O3	−43.9 (5)	C2—C1—S1—O2	23.0 (3)
C12—C13—C16—O3	137.8 (4)	C6—C1—S1—N1	92.0 (3)
C14—C13—C16—C17	133.8 (3)	C2—C1—S1—N1	−90.5 (3)
C12—C13—C16—C17	−44.5 (5)		

### Hydrogen-bond geometry (Å, º)

**Table:**

*D*—H···*A*	*D*—H	H···*A*	*D*···*A*	*D*—H···*A*
C8—H8···O1	0.93	2.38	2.953 (4)	120
C15—H15*A*···O2	0.97	2.22	2.808 (4)	118
C15—H15*B*···O3	0.97	2.38	3.062 (5)	127

## References

[bb1] Andreani, A., Granaiola, M., Leoni, A., Locatelli, A., Morigi, R., Rambaldi, M., Giorgi, G., Salvini, L. & Garaliene, V. (2001). *Anticancer Drug. Des.* **16**, 167–174. 11962514

[bb2] Bassindale, A. (1984). *The Third Dimension in Organic Chemistry*, ch. 1, p. 11. New York: John Wiley and Sons.

[bb3] Bruker (2004). *APEX2* and *SAINT*. Bruker AXS Inc., Madison, Wisconsin, USA.

[bb4] Chakkaravarthi, G., Marx, A., Dhayalan, V., Mohanakrishnan, A. K. & Manivannan, V. (2009). *Acta Cryst.* E**65**, o464–o465. 10.1107/S1600536809003493 PMC2968599 21582136

[bb5] Chakkaravarthi, G., Sureshbabu, R., Mohanakrishnan, A. K. & Manivannan, V. (2008). *Acta Cryst.* E**64**, o751. 10.1107/S1600536808007794 PMC2960915 21202141

[bb6] Grinev, A. N., Shevdov, V. L., Krichevskii, E. S., Romanova, O. B., Altukkhova, L. B., Kurilo, G. N., Andreeva, N. I., Golovina, S. M. & Mashkovskii, M. D. (1984). *Khim. Farm. Zh.* **18**, 159–163.

[bb7] Groom, C. R. & Allen, F. H. (2014). *Angew. Chem. Int. Ed.* **53**, 662–671. 10.1002/anie.201306438 24382699

[bb8] Porter, J. K., Bacon, C. W., Robbins, J. D., Himmelsbach, D. S. & Higman, H. C. (1977). *J. Agric. Food Chem.* **25**, 88–93. 10.1021/jf60209a043 1002941

[bb9] Rodriguez, J. G., Temprano, F., Esteban-Calderon, C., Martinez-Ripoll, M. & Garcia-Blanco, S. (1985). *Tetrahedron*, **41**, 3813–3823.

[bb10] Sheldrick, G. M. (1996). *SADABS*. University of Göttingen, Germany.

[bb11] Sheldrick, G. M. (2008). *Acta Cryst.* A**64**, 112–122. 10.1107/S0108767307043930 18156677

[bb12] Singh, U. P., Sarma, B. K., Mishra, P. K. & Ray, A. B. (2000). *Fol. Microbiol.* **45**, 173–176. 10.1007/BF02817419 11271828

[bb13] Spek, A. L. (2009). *Acta Cryst.* D**65**, 148–155. 10.1107/S090744490804362X PMC2631630 19171970

[bb14] Sundberg, R. J. (1996). *The Chemistry of Indoles*, p. 113. New York: Academic Press.

[bb15] Umadevi, M., Ramalingam, B. M., Yamuna, R., Mohanakrishnan, A. K. & Chakkaravarthi, G. (2014). *Acta Cryst.* E**70**, 466–468. 10.1107/S1600536814024064 PMC4257421 25552966

[bb16] Umadevi, M., Saravanan, V., Yamuna, R., Mohanakrishnan, A. K. & Chakkaravarthi, G. (2013). *Acta Cryst.* E**69**, o1781. 10.1107/S1600536813030730 PMC3885050 24454226

