# Supplementary figures and images for: Crystal structure of (2-bromo­methyl-1-phenyl­sulfonyl-1H-indol-3-yl)(phen­yl)methanone
Source: Acta Crystallogr E Crystallogr Commun. 2015 Jan 3;71(Pt 2):o86–7. doi: 10.1107/S2056989014028084 (PMC4384623; doi:10.1107/S2056989014028084)

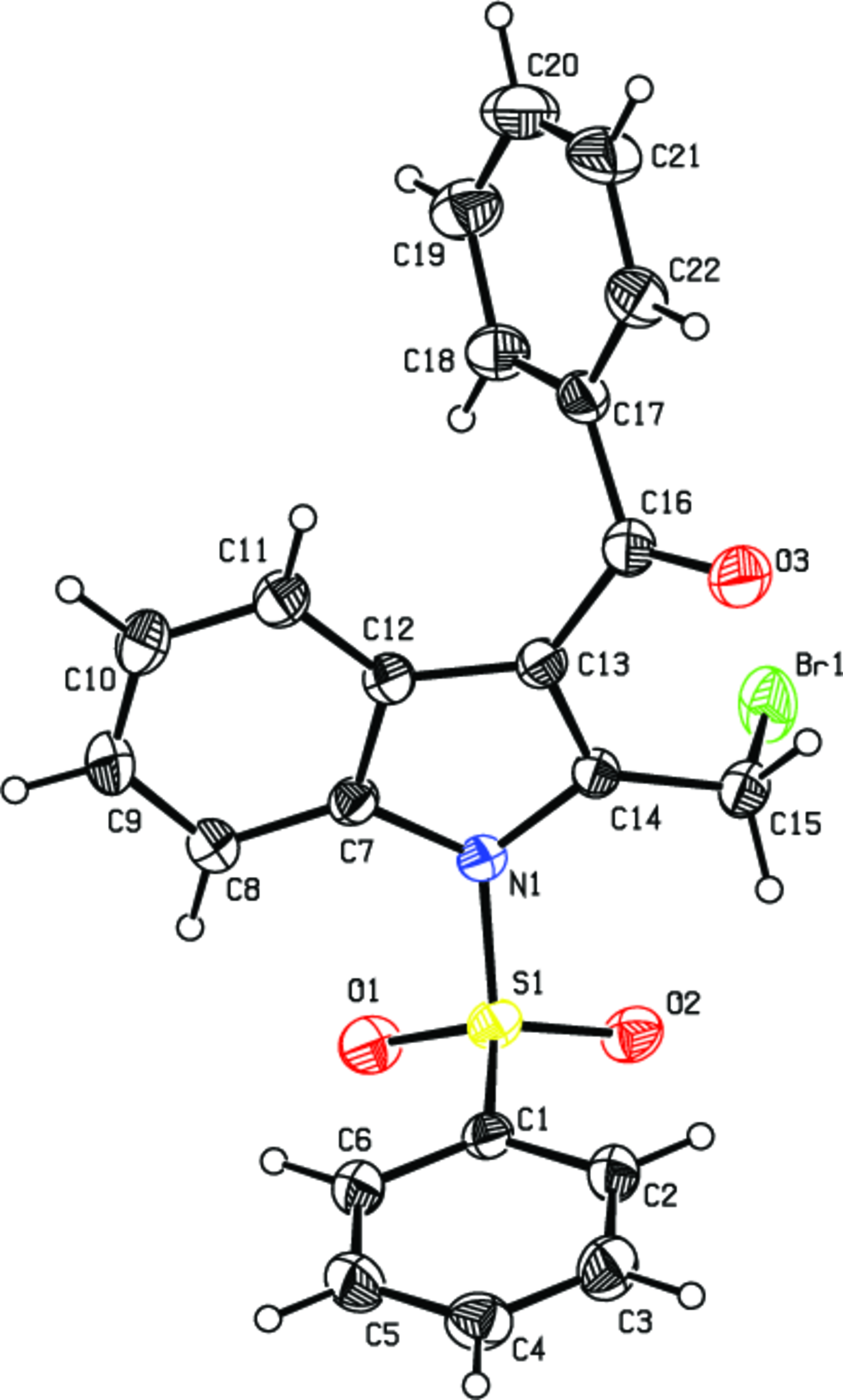

Supplement: Supplementary file 4 [file e-71-00o86-fig1.tif]

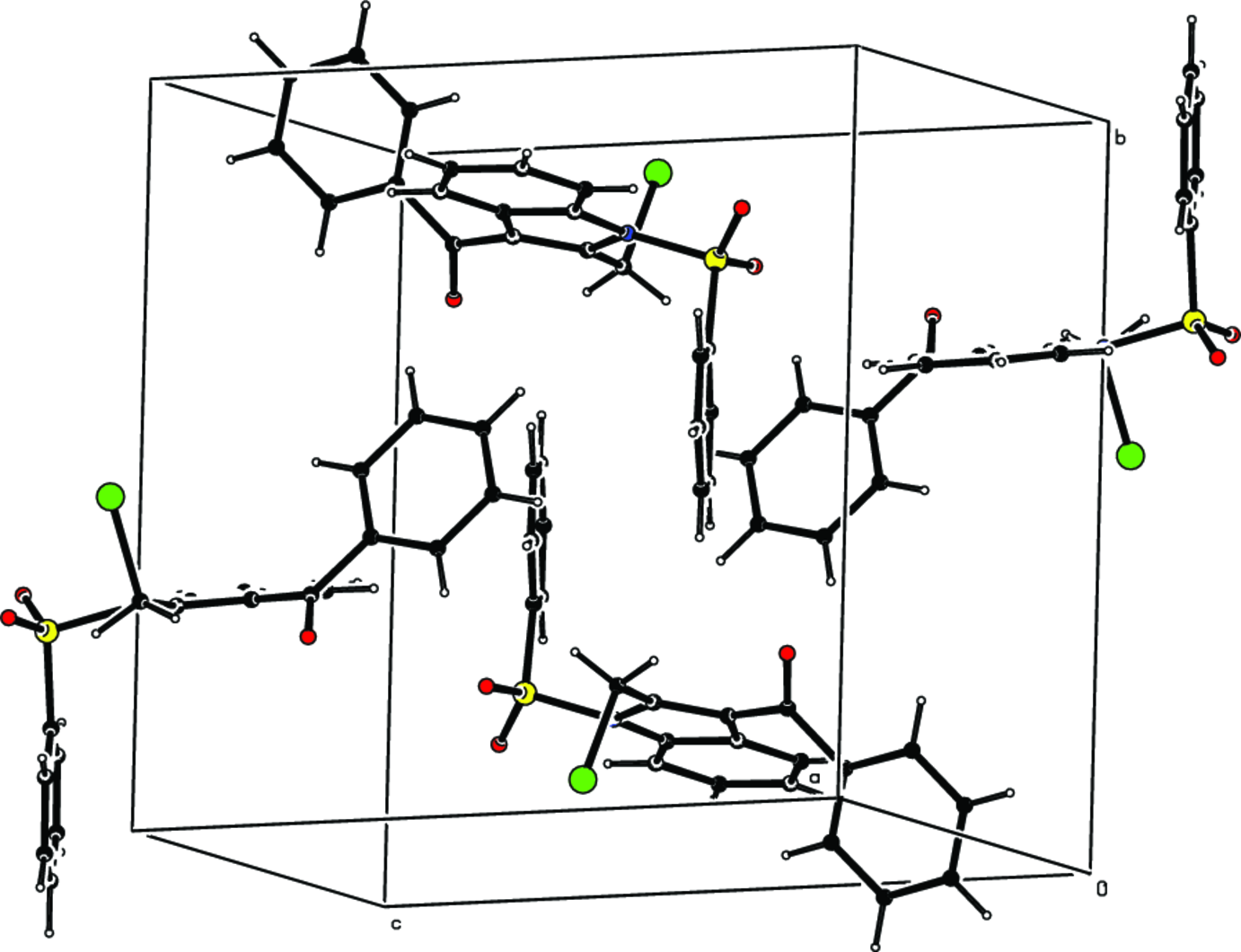

Supplement: Supplementary file 5 [file e-71-00o86-fig2.tif]
